# Barley β-Glucans-Containing Food Enhances Probiotic Performances of Beneficial Bacteria

**DOI:** 10.3390/ijms15023025

**Published:** 2014-02-20

**Authors:** Mattia P. Arena, Graziano Caggianiello, Daniela Fiocco, Pasquale Russo, Michele Torelli, Giuseppe Spano, Vittorio Capozzi

**Affiliations:** 1 S.A.F.E. Department, University of Foggia, Via Napoli 25, 71100 Foggia, Italy; E-Mails: mattiapia.arena@unifg.it (M.P.A.); graziano.caggianiello@unifg.it (G.C.); pasquale.russo@unifg.it (P.R.); vittorio.capozzi@gmail.com (V.C.); 2 Department of Clinical and Experimental medicine, University of Foggia, Via Pinto, 1, 71122 Foggia, Italy; E-Mail: daniela.fiocco@unifg.it; 3 Pastificio Attilio Matromauro Granoro s.r.l., Strada provinciale 231 km. 35,100-Corato (Bari), Italy; E-Mail: m.torelli@granoro.it

**Keywords:** barley β-glucans, probiotics, prebiotics, pasta, *Lactobacillus*

## Abstract

Currently, the majority of prebiotics in the market are derived from non-digestible oligosaccharides. Very few studies have focused on non-digestible long chain complex polysaccharides in relation to their potential as novel prebiotics. Cereals β-glucans have been investigated for immune-modulating properties and beneficial effects on obesity, cardiovascular diseases, diabetes, and cholesterol levels. Moreover, β-glucans have been reported to be highly fermentable by the intestinal microbiota in the caecum and colon, and can enhance both growth rate and lactic acid production of microbes isolated from the human intestine. In this work, we report the effects of food matrices containing barley β-glucans on growth and probiotic features of four *Lactobacillus* strains. Such matrices were able to improve the growth rate of the tested bacteria both in unstressed conditions and, importantly, after exposure to *in vitro* simulation of the digestive tract. Moreover, the effect of β-glucans-containing food on bacterial adhesion to enterocyte-like cells was analyzed and a positive influence on probiotic-enterocyte interaction was observed.

## 1. Introduction

The pivotal role of nutrition for maintaining a good state of health is a well-accepted notion. A correct diet can have preventive and curative effects on diseases and disorders of various origins, including obesity, phlogosis, immune dysfunctions, cancer and the detrimental consequences of aging [1–3].

Probiotic microorganisms are increasingly recognized for their beneficial effects on human health. Thus, microorganisms recognized as probiotics, mainly members of the *Lactobacillus* and *Bifidobacterium* genera, are increasingly being used in food preparations and for the development of novel functional foods [4,5].

Beyond the assessment of probiosis and the development of methods to identify new probiotic microorganisms, the concept of prebiosis, *i.e.*, the enhancement of probiotic function, has become as important as the notion of probiosis. Prebiosis consists in the selective stimulation of growth and/or activity of one or a limited number of beneficial microbial species in the gut microbiota, thus enhancing probiotic-deriving health benefits to the host [6,7]. Moreover, prebiotic properties have been related to improved efficiency in intestinal functions, mineral absorption, immune functions, and cancer prevention [8–15].

The gut microbiota comprises mostly anaerobic bacteria that need fermentative substrates to obtain metabolic energy for their growth and activity. Non-digestible food carbohydrates, including fibres, oligosaccharides, resistant starch, as well as proteins or peptides that escape from human digestion, can be utilized by microbes as a source of energy [16–18]. Several food components, including fructooligosaccharides (FOS) and galactooligosaccharides (GOS), have been shown to positively influence growth and metabolism of bifidobacteria and lactobacilli, as well as the overall composition of the gut microbiota, thus performing a prebiotic action. Among dietary fibres, β-glucans, together with other non-digestible food ingredients such as soybean oligosaccharides, lactosucrose, and isomalto-oligosaccharides, are currently being investigated to evaluate their potential prebiotic effects.

β-Glucans constitute the water-soluble fraction of several cereals and are stored in the cell walls of the aleurone and subaleurone layer of barley, oat, sorghum, triticale, wheat and rice [6,19]. In oat and barley, *i.e.*, cereals with high β-glucan levels, the content of such carbohydrates is in the range of 2–20 g and 3–8 g per 100 g of dry weight, respectively. Structurally, these cereal β-glucans are linear d-glucose polymers linked by either β(1,3) or β(1,4) glycosidic bonds, for which humans do not possess enzymes to split the link, and presenting side branches connected to the principal chain by β(1,2)- or (1,6)-glucopyranosyl substituents [20,21].

Other dietary sources of β-glucans comprise several species of fungi [22]. Fungal β-glucans can present linear β(1→3) or (1→6)-linked sugar units and branches of β(1→3) and β(1→6) glycosidic residues [22]. β-Glucans, are also among the most abundant polysaccharides produced by several bacteria, including *Pediococcus* and *Lactobacillus* species. β-Glucans of microbial origin usually present β(1,3) a linear glycosidic chain core and β(1,4) side branches structures [23].

Differences in the core linkage, chain branching, molecular weight, and solution conformation contribute to the different biological activity of β-glucans, which have been proven to possess immune-modulating properties [24,25] and beneficial effects on obesity, cardiovascular diseases, diabetes, and cholesterol levels [26–29]. Moreover, β-glucans have been shown to be highly fermentable by the intestinal microbiota of caecum and colon, and could enhance the growth rate and the lactic acid production of microbes isolated from human intestine [8,30]. In this work, we examine the effects of barley β-glucans on four *Lactobacillus* strains, evaluating growth capabilities and some probiotic features, including tolerance to *in vitro* simulated oro-gastrointestinal (OGI) stresses. In this regard, the potential influence of two different food matrices containing β-glucans, *i.e.*, β-glucans enriched pasta and barley flour, was studied. Furthermore, *in vitro* adhesion assays on human intestinal cells were performed with and without β-glucans-containing matrices, in order to understand whether barley β-glucans could improve the intestinal colonization ability of probiotics.

## 2. Results and Discussion

### 2.1. Food Matrices Containing β-Glucans Stimulate Bacterial Growth

**Figure 1.**
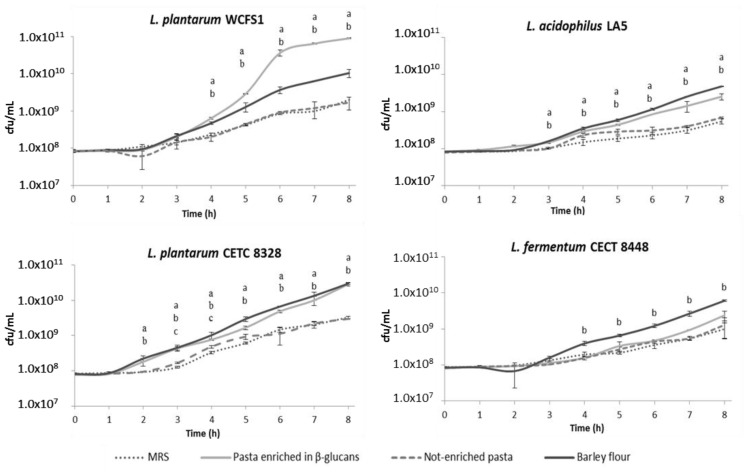
Growth curves of *L. plantarum* WCFS1, *L. acidophilus* LA5, *L. plantarum* CETC 8328 and *L. fermentum* CECT 8448. Bacteria were grown in MRS and MRS supplemented with different food matrices (not-enriched pasta, β-glucans enriched pasta and barley flour). Values represent mean ± standard deviation of three different experiments. Statistical analysis was carried out by Student’s *t*-test and significant differences are relative to MRS (positive control). ^a^ *p* < 0.005 for samples cultivated in MRS supplemented with β-glucans enriched pasta; ^b^ *p* < 0.005 for samples cultivated in MRS supplemented with barley flour; ^c^ *p* < 0.005 for samples cultivated in MRS supplemented with not-enriched pasta.

In order to identify the prebiotic potential of barley β-glucans, the effects of two different β-glucans-enriched matrices were investigated on reference probiotics, *i.e.*, *Lactobacillus acidophilus* LA5 and *Lactobacillus plantarum* WCFS1, and strains of *Lactobacillus plantarum* CETC 8328 and *Lactobacillus fermentum* CECT 8448, previously characterized [31,32]. The bacterial growth rate was monitored both by optical density (OD_600 nm_) (data not shown) and by CFU counting on agar plates (Figure 1). CFU analysis provided the best method to monitor microbial growth as the presence of the food matrix determined a considerable turbidity of the growth media that could bias spectrophotometric analyses. The results showed that food matrices containing β-glucans (β-glucans enriched pasta and barley flour) stimulated the growth of the bacterial strains analysed to a greater extent than traditional pasta and MRS alone.

The ability of β-glucans to influence the growth of microorganisms was strain-selective, as a significantly higher stimulation was observed for *L. plantarum* WCFS1 and *L. plantarum* CETC 8328 than *L. acidophilus* LA5 and *L. fermentum* CECT 8448. Furthermore, differences in growth were observed on the different β-glucan enriched matrices. For example, the growth of *L. fermentum* CECT 8448 was only affected by barley flour rather than by β-glucans enriched pasta, while the growth of *L. plantarum* WCFS1, *L. plantarum* CETC 8328 and *L. acidophilus* LA5, was positively influenced by both barley flour and β-glucans enriched pasta. Interestingly, the growth rate of *L. plantarum* WCFS1 was much more improved by β-glucans enriched pasta than by barley flour, probably because of the differences in the β-glucans availability due to food processing. In fact, prebiotic substrates comprise a heterogeneous complex of molecules that can promote the growth and the activity of specific strains of bacteria as they do not influence any metabolic activity of other microorganisms. Thus, the probiotic strain considered, the type of prebiotic and their bioavailability, the manufacture processes, and the solubility, polymerization and viscosity within the food matrix constitute variables that can be determined to have the best potential synergy between prebiosis and probiosis [33–36]. Indeed, several dietary carbohydrates, including β-glucans, were found unable to influence the growth of a range of probiotic and intestinal bacterial strains belonging to *Lactobacillus* and *Bifidibacterium* genera [33]. However, considering the differences between β-glucans from cereals and those of microbial origin, other authors reported a positive effect of β-glucans extracted from *Pediococcus parvulus* 2.6 on the growth of *L. plantarum*, suggesting the utilization of such carbohydrates as a carbon source [37]. Likewise, commercially available β-glucans hydrolysate and β-glucans concentrate from barley, have been shown to enhance the growth rate of *Bifidobacterium animalis* and *Lactobacillus casei* in a basal culture medium, although the positive influence of β-glucans concentrate was lower than that of β-glucans hydrolysate. The minor effect of β-glucans concentrate was likely correlated to the poor solubility, reflecting the importance of the compound bioavailability within the matrix carrier [38].

Furthermore, cereal β-glucan-rich fractions obtained by debranning were showed suitable for lactic acid bacteria as fermentable substrates to support their growth [30].

To sum up, we exposed four selected probiotic strains to different food matrices to investigate the growth rate of bacteria. Our results, considering the complexity of the food matrix that contain proteins, vitamins, minerals and also β-glucans (*i.e.*, β-glucans enriched pasta and barley flour), indicated that the food matrix containing β-glucans have a positive effect on the growth of the selected probiotic strains compared to the not-enriched food matrix.

### 2.2. Food Matrix Improves the Tolerance to Oro-Gastrointestinal Transit Simulation

**Figure 2.**
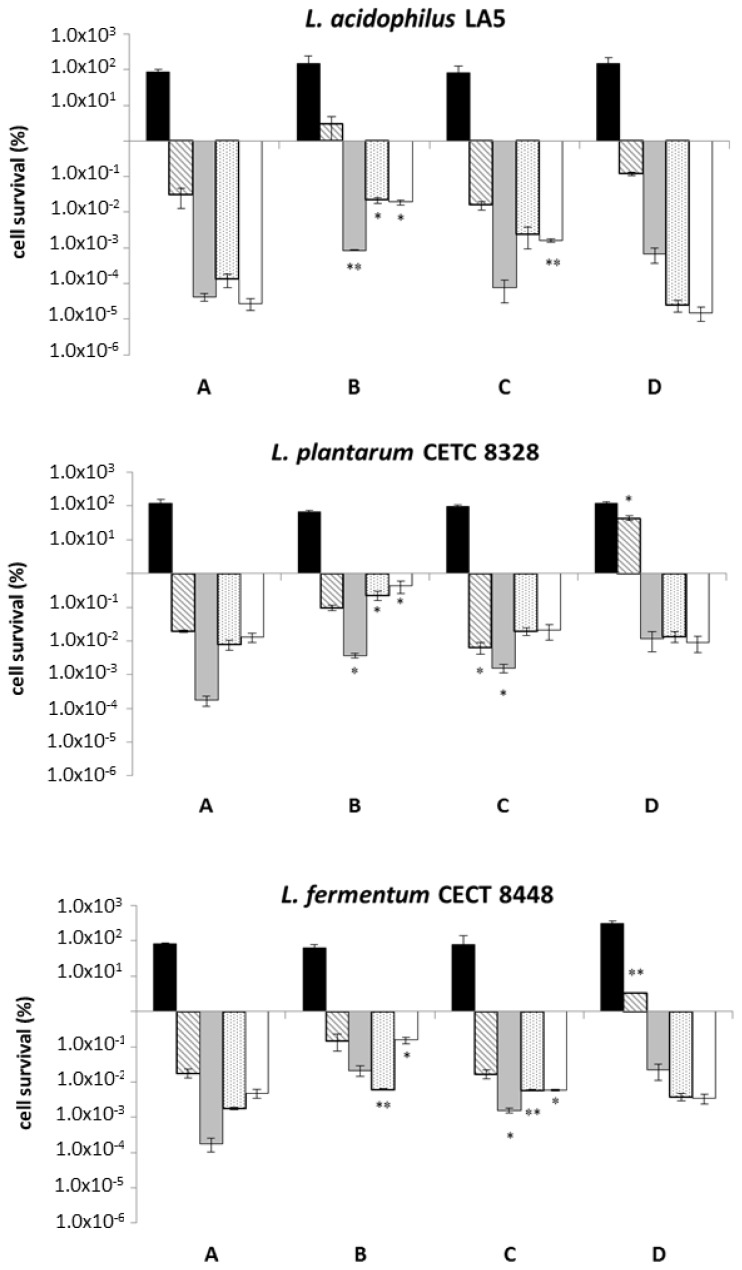
Percentage of cell survival after *in vitro* oro-gastrointestinal tract simulation, including oral ■, gastric (pH 2.0 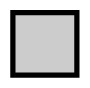 and pH 3.0 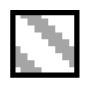), and intestinal (small 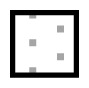 and large intestine □) stresses conveying microorganisms in saline solution (negative control) and three different food matrices (not-enriched pasta; β-glucans enriched pasta; barley flour). Values represent mean ± standard deviation of three different experiments. Statistical analysis was carried out by Student’s *t*-test (* *p* < 0.05 and ** *p* < 0.005) and significant differences are relative to OGI transit using saline solution as a carrier. Cell survival is expressed as a percentage (in logarithmic scale) relative to the untreated control sample (1.00 × 10^2^ corresponds to 100%, 1.00 × 10^6^ corresponds to 0.000001%).

All strains were exposed to an *in vitro* model mimicking the human OGI tract [38]. The ability of *L. acidophilus* LA5, *L. plantarum* CETC 8328 and *L. fermentum* CECT 8448 to survive OGI stresses is reported in Figure 2. The percentage of survival for *L. plantarum* WCFS1 is not shown, as the ability of this strain to resist to OGI conditions was previously examined [39]. The bacterial cultures were resuspended in saline solution, not-enriched pasta, β-glucans enriched pasta and barley flour as carriers, with the aim to investigate the potential effects of the food matrix and β-glucans on bacterial resistance to OGI stresses. Overall, the viability of all the analyzed bacterial strains was negatively affected by saline solution. In particular, viability was significantly reduced under gastric conditions (pH 3.0 and pH 2.0). Indeed, the percentage of survival was reduced by about 4 and 6 Log units under pH 3.0 and 2.0, respectively, with no significant differences between the tested strains. These findings are in agreement with those reported by several authors, according to whom the low pH stress is usually the hardest obstacle for survival of probiotic bacteria [39–41]. When not-enriched pasta was used as a carrier, the survival of all considered strains was less impaired, probably due to the protective effect of such matrix. Specifically, we found a reduction of bacteria survival by around 2 Log units (*L. acidophilus* LA5) and 3 Log units (*L. plantarum* CETC 8328 and *L. fermentum* CECT 8448) after gastric stress (pH 3.0). The subsequent acidic stress at pH 2.0, often considered the strongest environmental challenge for probiotics, caused a decrease of cell survival of about 4–5 Log units. In the presence of not-enriched pasta as a carrier, we observed reductions by 3 Log units for *L. plantarum* CETC 8328 and 4 Log units for *L. acidophilus* LA5 and *L. fermentum* CECT 8448, thus indicating a greater tolerance to intestinal conditions (small and large intestine stresses). We noticed a greater capability of probiotic strains to survive under stress conditions when β-glucans enriched pasta was used as a carrier compared to saline solution, although no significant difference was observed between not-enriched and β-glucans enriched pasta. Lastly, barley flour efficiently protected microorganisms during the OGI simulated transit and this was particularly apparent under acidic conditions at pH 3.0, with reduction of cell survival by 1 (*L. plantarum* CETC 8328), 1.5 (*L. fermentum* CECT 8448) and 3 Log units (*L. acidophilus* LA5). Overall, our results suggest that food matrices enhance the tolerance of probiotics to the OGI transit, confirming results previously reported by other authors [39,42,43]. Conversely, no significant influence on bacterial tolerance to the tested OGI stresses could be attributed to β-glucans as the rates of survival were quite similar in barley flour and in both types of pasta, regardless of the addition of such carbohydrates.

### 2.3. β-Glucans Stimulate Bacterial Growth after OGI Stress

The tolerance to OGI stresses is one of the most important attributes for probiotics, as are the capability to colonize the intestine, to resume the metabolic activity and to exert beneficial action [43,44]. In this context, we investigated the recovery of bacterial growth after exposure to OGI tract simulation. The differences of growth rate in relation to the diverse food matrices (not-enriched pasta, β-glucans enriched pasta and barley flour) were examined and compared to the negative control (saline solution).

**Figure 3.**
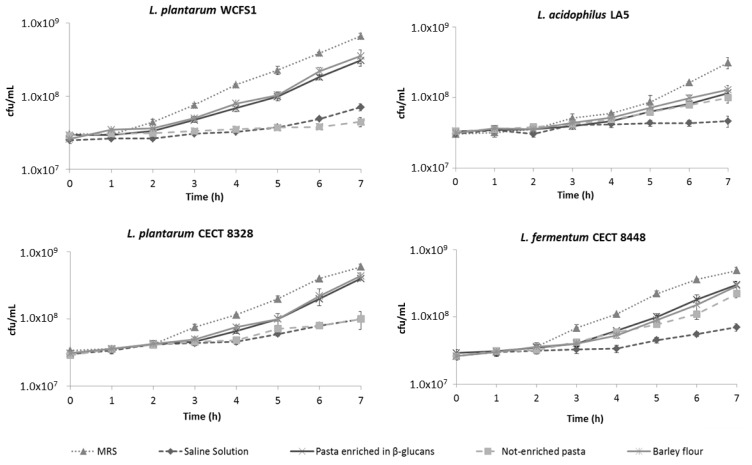
Growth curves of *L. plantarum* WCFS1, *L. acidophilus* LA5, *L. plantarum* CETC 8328 and *L. fermentum* CECT 8448, grown in MRS after exposure to oro-gastrointestinal stresses using saline solution and different food matrix (not-enriched pasta, β-glucans enriched pasta and barley flour). Values represent mean ± standard deviation of three different experiments. Statistical analysis (data not shown) was carried out by Student’s *t*-test (see Results and Discussion section).

Figure 3 shows the growth curves obtained by monitoring the CFU of bacterial cultures after the entire OGI tract simulation. All the strains exposed to OGI simulation using both β-glucans enriched pasta and barley flour as carriers exhibited faster growth after the stresses were removed. In all cases, the higher growth rate meant a significant reduction of the latent phase compared to negative control. In the case of *L. plantarum* strains WCFS1 and CETC 8328, the bacterial growth was positively affected by the two matrices containing β-glucans, with no significant differences between β-glucans enriched pasta and barley flour. In contrast, we observed a minor effect on bacterial recovery when not-enriched pasta was used. In the case of *L. acidophilus* LA5 and *L. fermentum* CECT 8448, we did not detect any significant variation between the growth rates of bacteria after the OGI simulation either in presence of not-enriched pasta or β-glucans enriched pasta and barley flour.

Overall, these data suggest that the presence of β-glucans in the food matrices may promote the recovery of probiotic growth, probably based on the selective metabolic utilization of β-glucans from barley, especially by those bacterial strains belonging to *L. plantarum* species.

### 2.4. β-Glucans Influence Probiotic Adhesion to Caco-2 Cells

**Figure 4.**
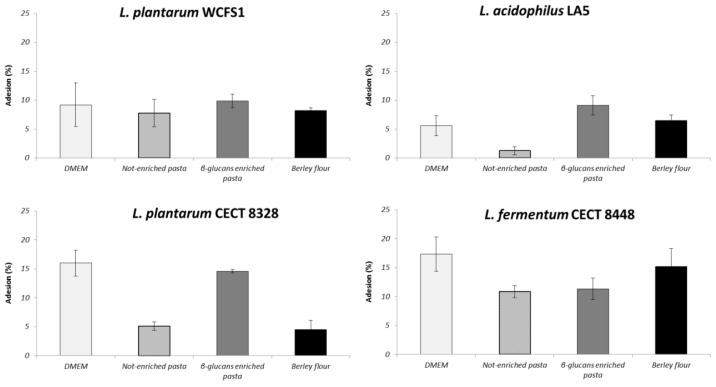
Adhesion of *L. plantarum* WCFS1, *L. acidophilus* LA5, *L. plantarum* CETC 8328 and *L. fermentum* CECT 8448 to Caco-2 cells in the presence of DMEM medium (positive control), not-enriched pasta, β-glucans enriched pasta and barley flour. Values represent mean ± standard deviation of three different experiments.

Adhesion capability is one of the most important features of probiotic microorganisms as it guarantees colonization, persistence, and proliferation of beneficial bacteria within the intestinal environment of the host. The ability to adhere to human enteric Caco-2 cells was investigated for all probiotic strains (Figure 4). Firstly, we analyzed the adhesion to Caco-2 cells using only the culture cell medium (DMEM, positive control) and the percentages of adhesion were found to be around 9%, 6%, 16%, 17% for *L. plantarum* WCFS1, *L. acidophilus* LA5, *L. plantarum* CETC 8328 and *L. fermentum* CECT 8448, respectively. Subsequently, all strains were incubated with Caco-2 cells in the presence of the different food matrices (not-enriched pasta, β-glucans enriched pasta and barley flour). The ability of *L. plantarum* WCFS1, *L. acidophilus* LA5, *L. plantarum* CETC 8328 and *L. fermentum* CECT 8448 to adhere to intestinal cells in the presence of not-enriched pasta and β-glucans enriched pasta was estimated around 8%, 1%, 5%, and 11% and 10%, 9%, 15%, and 11%, respectively. When co-incubating probiotic bacteria and barley flour, the percentages of adhesion were approximately 8%, 6%, 5% and 15% for *L. plantarum* WCFS1, *L. acidophilus* LA5, *L. plantarum* CETC 8328 and *L. fermentum* CECT 8448, respectively. Therefore, our results suggest that the adhesion properties of probiotic bacteria are strictly dependent on the food matrix. As reported in Figure 4, the presence of food matrices determined a general decrease in the percentage of adhesion compared to control condition. However, *L. acidophilus* LA5 exhibited a greater ability to adhere when incubated with β-glucans enriched pasta and barley flour rather than with traditional pasta and, for almost all strains, the presence of β-glucans in the pasta seemed to ameliorate adhesion. A positive effect of β-glucans on the adhesion of probiotic strains was also reported by others authors that observed a significant enhancement of *L. plantarum* adherence to intestinal cells in the presence of purified β-glucans of bacterial origin [37]. At the same time, the effect of the matrix type resulted to be quite strain dependent, as differences were observed between the strains and the matrices used. For example, *L. fermentum* CECT 8448 was the most adherent strain when not-enriched pasta and barley flour were tested. By contrast, the highest adhesion level was exhibited by *L. plantarum* CETC 8328 when β-glucans enriched pasta was used.

The negative effect of the presence of a complex food matrix on microbial adhesion ability is in agreement to previous studies. For instance, milk was reported to reduce probiotic adhesion to Caco-2 cells [45]. We speculate that pasta and flour matrices could interfere with the adhesion process by moderately preventing the probiotic-enterocyte contacts, probably because of the physical entrapment of bacteria within the food substrate. Ouwehand and Salminen [45] suggested that the negative effect of food matrix on probiotic-enterocyte interaction could depend on physical entrapment within the matrix itself, on specific or non-specific binding to adhesins, or on the steric hindrance of adhesins Moreover, β-glucans have been shown to form viscous solutions in water and in human intestine [46–48]. Although, such characteristics have been correlated to beneficial effects on serum cholesterol and postprandial blood glucose levels for humans [47], viscosity could conceptually determine a partial obstacle for probiotic bacteria to recognize the adhesion sites on the enterocyte surface. Interestingly, the entrapment of probiotics within the food substrate could also account for the food protective effect observed during the OGI simulation. Indeed, although the probiotic-food matrices combinations resulted in a partial disadvantage for the adhesion to enterocytes, such combinations, on the other hand, are likely to increase the percentage of microbial survival to OGI challenges, thus enhancing the chance for them to reach the intestine in a viable and effective status.

## 3. Experimental Section

### 3.1. Bacterial Strains, Human Cells and Growth Conditions

The lactobacilli used in this work were *L. plantarum* WCFS1, *L. acidophilus* LA5, described as probiotic strains [49–52], and the riboflavin over-producing strains *L. plantarum* CETC 8328 and *L. fermentum* CECT 8448, which were previously selected as spontaneous roseoflavin-resistant isolates and characterized for having probiotic features [31,32]. The strains were propagated in de Man, Rogosa, Sharpe (MRS, Oxoid, UK) (pH 6.2) and incubated at 30 °C. For *L. acidophilus* LA5, MRS medium was supplemented with 0.1% Tween and 0.05% l-cysteine (Merck, Darmstad, Germany) and incubation was performed at 37 °C. Human adenocarcinoma colon cells (Caco-2) (Sigma-Aldrich, St. Louis, MO, USA) were seeded at a concentration of 1.2 × 10^4^ cells/well in 96-wells plates and grown in Dulbecco’s Modified Eagle Medium (DMEM, Sigma-Aldrich, St. Louis, MO, USA) supplemented with 10% (*v*/*v*) heat inactivated fetal bovine serum (FBS), 2 mM l-glutamine, 100 U/mL penicillin and 100 μg/mL streptomycin, at 37 °C, in atmosphere containing 5% CO_2_. In order to obtain differentiated and polarized enterocyte-like monolayers, Caco-2 cells were cultured for a period of 15 days, during which time the supplemented medium was replaced three times per week. Twenty-four hours prior to the adhesion assay, the growth medium was replaced with absolute DMEM, without any supplements.

### 3.2. Influence of Different Food Matrices on Bacterial Growth

Not-enriched pasta (control), β-glucans enriched pasta (3 g of β-glucans per 100 g of pasta, obtained mixing flours of barley and durum wheat), and flour of barley (12 g of β-glucans per 100 g of flour) were kindly provided by Granoro srl (Corato, Italy). Microorganisms were grown at their optimal growth temperatures in MRS and in all the samples analyzed (see below). The bacterial growth was monitored by optical density (OD_600 nm_) (only for MRS medium) and by counting CFU on agar plates (for MRS and all matrices).

### 3.3. Oro-Gastrointestinal Tolerance Assay

Bacteria were cultured until they reached the mid-exponential phase (OD_600 nm_ 1, corresponding to a concentration of 2–8 × 10^8^ CFU/mL, according to the specific strain), then were centrifuged (2000× *g*, 10 min) and resuspended in three different carrier matrices: (i) NaCl 8.5 g/L; (ii) not-enriched pasta; (iii) β-glucans enriched pasta; and (iv) barley flour containing 12 g of β-glucans per 100 g of flour. Pasta was weighed (20 g) (both control and enriched in β-glucans) and boiled in 200 mL of water for 10 min. Subsequently, the cooking water was eliminated and pasta was homogenized with 200 mL of sterilized saline solution (NaCl 8.5 g/L) using a homogenizer with sterilized supports. Finally, the homogenized pasta was further diluted with saline solution in order to have a final concentration of 33 mg/mL of pasta (corresponding to 1 mg/mL of β-glucans for the β-glucans enriched pasta matrix). Similarly, 0.008 g/mL of barley flour were mixed and boiled in saline solution and then, under sterile conditions, the final volume was adjusted in order to have a final concentration of 1 mg/mL of β-glucans. The simulated gastrointestinal transit was performed as already described [39] but, in the present study, only selected steps corresponding to the oral compartment, gastric sectors at pH 3.0 and 2.0 and subsequent intestinal stresses, *i.e.*, small and large intestine, were considered. Dilutions from control and treated samples of each strain were plated on MRS agar, CFU were counted and percent survival was determined with respect to unstressed samples (positive control). After the entire oro-gastrointestinal tract simulation, *i.e.*, after the large intestine stress deriving from gastric compartment at pH 2.0, samples were centrifuged (2000× *g*, 10 min), in order to remove the OGI solutions, and appropriately diluted in fresh MRS. Microorganisms were grown 37 °C and growth was monitored by counting CFU on agar plates.

### 3.4. *In Vitro* Adhesion Assay

Adhesion assays were performed on Caco-2 cell monolayers according to Russo *et al.* [37]. All bacterial strains were cultured until they reached the mid-exponential phase (OD_600 nm_ 1), then were centrifuged (2000× *g*, 10 min) and resuspended in (i) absolute DMEM; (ii) DMEM supplemented with not-enriched pasta; (iii) DMEM supplemented with β-glucans enriched pasta; and (iv) DMEM added with flour of barley. The final concentrations of each supplemented matrix were the same as the previous trials. Microorganism and Caco-2 cells (ratio 1000:1) were co-incubated for 1 h, at 37 °C, with 5% CO_2_. The percentage of adhesion by lactobacilli was determined by plating serial dilutions of the bacterial suspensions from control and test wells on MRS agar and subsequent CFU counting [37].

### 3.5. Statistical Analysis

Data were analyzed by Student’s *t*-test using the IBM SPSS Statistics 21.0 software program (IBM, Armonk, NY, USA). *p* < 0.05 and *p* < 0.005 were considered as statistically significant.

## 4. Conclusions

In conclusion, we found that β-glucans from barley were able to improve the growth rate of the tested probiotic bacteria both in unstressed conditions and more importantly after exposure to *in vitro* digestive tract simulation, with a selective influence on *L. plantarum* species. Notably, the stimulation of metabolism of beneficial microorganisms, as well as the selectivity, is one of the key features claimed for prebiotic food products. Considering that when probiotic bacteria are ingested, they have to pass through the entire human digestive tract, specific strains that tolerate low pH, bile salts, and digestive enzymes well, are strongly preferred to be used as probiotics. In this context, we reported that food matrices, including those containing β-glucans, enhanced the oro-gastrointestinal stress tolerance by probiotic strains. Although survival in the OGI transit, as determined by CFU analysis, was substantially unaffected by the presence of β-glucans in the carrier matrix, it is interesting to note that growth recovery after such stresses was, for some specific strains, significantly faster when β-glucans-containing matrices were used. By analyzing the influence of the different food-matrices on probiotic adhesion, we found that they generally affected the probiotic-enterocytes interaction in a negative way. However and interestingly, the adverse influence on adherence ability was alleviated when β-glucans-containing matrices were used, *i.e.*, barley flour and enriched pasta.

The ability of microorganisms to produce exopolysaccharides, such as β-glucans, was presented to be a desirable characteristic as it contributes to enhance bacterial survival and increase the concentration of microorganisms that can reach and colonize the intestine [37,40,45]. However, probiotics do not always themselves produce adequate amounts of β-glucans. Therefore, the preparation of prebiotic food formulations specifically enriched in β-glucans should gain considerable attention, consistent with a positive effect of β-glucans on food vehicled-probiotics and on the human microbiota composition. Clearly, the development of β-glucans-containing food as a suitable vehicle for friendly microorganisms, might even utilize their probiotic performances. Obviously, because food processing, including cooking, freezing, drying, and storing, could modify molecular weight, solubility, polymerization, viscosity and extractability of β-glucans within the food matrix, and, consequently, their correlated biological activities [33–36], it becomes crucial to better investigate the link between β-glucans, food substrate, manufacture processes and storage, to find and select the best conditions that enable and improve the prebiotic effects of such compounds.

## References

[1] Lam K.L., Cheung C.K. (2013). Non-digestible long chainbeta-glucans as novel prebiotics. Bioact. Carbohydr. Diet. Fibers.

[2] Collado M.C., Isolauri E., Salminen S., Sanz Y. (2009). The impact of probiotic on gut health. Curr. Drug Metab.

[3] Schrezenmeir J., de Vrese M. (2001). Probiotics, prebiotics, and synbiotics-approaching a definition. Am. J. Clin. Nutr.

[4] Food and Agriculture Organization of United Nations and World Health Organization Working Group (FAO/WHO) (2002). Guidelines for the Evaluation of Probiotics in Food.

[5] Vasilijevic T., Shah N.P. (2007). Fermented Milk: Health Benefits beyond Probiotic Effect. Handbook of Food Products Manufacturing.

[6] Mayo B., van Sinderen D., Ventura M. (2008). Genome analysis of food grade lactic acid-producing bacteria: From basics to applications. Curr. Genomics.

[7] Roberfroid M., Gibson G.R., Hoyles L., McCartney A.L., Rastall R., Rowland I., Wolvers D., Watzl B., Szajewska H., Stahl B. (2010). Prebiotic effects: Metabolic and health benefits. Br. J. Nutr.

[8] Blaut M. (2002). Relationship of prebiotics and food to intestinal microflora. Eur. J. Nutr.

[9] Dass N.B., John A.K., Bassil A.K., Crumbley C.W., Shehee W.R., Maurio F.P., Moore G.B.T., Taylor C.M., Sanger G.J. (2007). The relationship between the effects of short-chain fatty acids on intestinal motility *in vitro* and GPR43 receptor activation. Neurogastroenterol. Motil.

[10] Xiong Y., Miyamoto N., Shibata K., Valasek M.A., Motoike T., Kedzierski R.M., Yanagisawa M. (2004). Short-chain fatty acids stimulate leptin production in adipocytes through the G protein-coupled receptor GPR41. Proc. Natl. Acad. Sci. USA.

[11] Williams E.A., Coxhead J.M., Mathers J.C. (2003). Anti-cancer effects of butyrate: use of micro-array technology to investigate mechanisms. Proc. Nutr. Soc.

[12] Scheppach W. (1996). Treatment of distal ulcerative colitis with short-chain fatty acid enemas a placebo-controlled trial. Dig. Dis. Sci.

[13] Artis D. (2008). Epithelial-cell recognition of commensal bacteria and maintenance of immune homeostasis in the gut. Nat. Rev. Immunol.

[14] Nilsson N.E., Kotarsky K., Owman C., Olde B. (2003). Identification of a free fatty acid receptor, FFA2R, expressed on leukocytes and activated by short-chain fatty acids. Biochem. Biophys. Res. Commun.

[15] Le Poul E., Loison C., Struyf S., Springael J.-Y., Lannoy V., Decobecq M.-E., Brezillon S., Dupriez V., Vassart G., van Damme J. (2003). Functional characterization of human receptors for short chain fatty acids and their role in polymorphonuclear cell activation. J. Biol. Chem.

[16] Topping D.L., Clifton P.M. (2001). Short-chain fatty acids and human colonic function: Roles of resistant starch and nonstarch polysaccharides. Physiol. Rev.

[17] Lupton J. (2004). Microbial degradation products influence colon cancer risk: The butyrate controversy. J. Nutr.

[18] Macfarlane G.T., Gibson G.R., Cummings J.H. (1992). Comparison of fermentation reactions in different regions of the human colon. J. Appl. Microbiol.

[19] Holtekjølen A.K., Uhlen A.K., Bråthen E., Sahlstrøm S., Knutsen S.H. (2006). Contents of starch and non-starch polysaccharides in barley varieties of different origin. Food Chem.

[20] El Khoury D., Cuda C., Luhovyy B.L., Anderson G.H. (2012). Beta Glucan: Health benefits in obesity and metabolic syndrome. J. Nutr. Metab.

[21] Barsanti L., Passarelli V., Evangelista V., Frassanito A.M., Gualtieri P. (2011). Chemistry, physico-chemistry and applications linked to biological activities of β-glucans. Nat. Prod. Rep.

[22] Synytsya A., Novák M. (2013). Structural diversity of fungal glucans. Carbohydr. Polym.

[23] Godfrey C., Wing C., Daniel S. (2009). The effects of β-glucan on human immune and cancer cells. J. Hematol. Oncol.

[24] Murphy E.A., Davis J.M., Brown A.S., Carmichael M.D., Carson J.A., van Rooijen N., Ghaffar A., Mayer E.P. (2008). Benefits of oat β-glucan on respiratory infection following exercise stress: Role of lung macrophages. Am. J. Physiol.

[25] Volman J.J., Mensink R.P., Ramakers J.D., de Winther M.P., Carlsen H., Blomhoff R., Buurman W.A., Plat J. (2010). Dietary (1→3), (1→4)-β-d-glucans from oat activate nuclear factor-kappa B in intestinal leukocytes and enterocytes from mice. Nutr. Res.

[26] Slavin J.L. (2005). Dietary fiber and body weight. Nutrition.

[27] Jensen M.K., Koh-Banerjee B., Hu F.B., Franz M., Sampson L., Grønbæk M., Rimm E.B. (2004). Intakes of whole grains, bran, and germ and the risk of coronary heart disease in men. Am. J. Clin. Nutr.

[28] Brennan C.S. (2005). Dietary fibre, glycaemic response, and diabetes. Mol. Nutr. Food Res.

[29] Önning G., Wallmark A., Persson M., Åkesson B., Elmståhl S., Öste R. (1999). Consumption of oat milk for 5 weeks lowers serum cholesterol and LDL cholesterol in freeliving men with moderate hypercholesterolemia. Ann. Nutr. Metab.

[30] Kedia G., Vázquez J.A., Pandiella S.S. (2008). Evaluation of the fermentability of oat fractions obtained by debranning using lactic acid bacteria. J. Appl. Microbiol.

[31] Capozzi V., Menga V., Digesù A.M., de Vita P., van Sinderen D., Cattivelli L., Fares C., Spano G. (2011). Biotechnological production of vitamin B2-enriched bread and pasta. J. Agric. Food Chem.

[32] Russo P., Capozzi V., Arena M.P., Spadaccino G., Dueñas M.T., López P., Fiocco D., Spano G. (2014). Riboflavin overproducing strains of *Lactobacillus fermentum* for riboflavin enriched bread. Appl. Microbiol. Biotechnol.

[33] Crittenden R., Karppinen S., Ojanen S., Tenkanen M., Fagerström R., Mättö J., Saarela M., Mattila-Sandholm T., Poutanen K. (2002). *In vitro* fermentation of cereal dietary fibre carbohydrates by probiotic and intestinal bacteria. J. Sci. Food Agric.

[34] Johansson L., Tuomainen P., Anttila H., Rita H., Virkki L. (2007). Effect of processing on the extractability of oat β-glucan. Food Chem.

[35] Tosh S.M., Brummer Y., Wolever T.M.S., Wood P.J. (2008). Glycemic response to oat bran muffins treated to vary molecular weight of β-glucan. Cereal Chem.

[36] Åman P., Rimsten L., Andersson R. (2004). Molecular weight distribution of β-glucan in oat-based foods. Cereal Chem.

[37] Russo P., López P., Capozzi V., Fernández de Palencia P., Dueñas M.T., Spano G., Fiocco D. (2012). β-Glucans improve growth, viability and colonization of probiotic microorganisms. Int. J. Mol. Sci.

[38] Su P., Henriksson A., Mitchell H. (2007). Selected prebiotics support the growth of probiotic mono-cultures *in vitro*. Food Microbiol.

[39] Bove P., Russo P., Capozzi V., Gallone A., Spano G., Fiocco D. (2013). *Lactobacillus plantarum* passage through an oro-gastro-intestinal tract simulator: Carrier matrix effect and transcriptional analysis of genes associated to stress and probiosis. Microbiol. Res.

[40] Fernández de Palencia P., Werning M.L., Sierra-Filardi E., Dueñas M.T., Irastorza A., Corbí A.L., López P. (2009). Probiotic properties of the 2-Substituted (1,3)-β-d-glucan-producing bacterium *Pediococcus parvulus* 2–6. Appl. Environ. Microbiol.

[41] Both E., György É., Kibédi-Szabó C.Z., Tamás É., Ábrahám B., Miklóssy I., Lányi S. (2010). Acid and bile tolerance, adhesion to epithelial cells of probiotic microorganisms. UPB Sci. Bull. B.

[42] Bezkorovainy A. (2001). Probiotics: Determinants of survival and growth in the gut. Am. J. Clin. Nutr.

[43] De Vrese M., Schrezenmeir J. (2008). Probiotics, prebiotics, and synbiotics. Food Biotechnol.

[44] Morelli L. (2000). *In vitro* selection of probiotic lactobacilli: A critical appraisal. Curr. Issues Intest. Microbiol.

[45] Ouwehand A.C., Salminen S. (2003). *In vitro* adhesion assays for probiotics and their *in vivo* relevance: A review. Microb. Ecol. Health Dis.

[46] Stack H.M., Kearney N., Stanton C., Fitzgerald G.F., Ross R.P. (2010). Association of β-glucan endogenous production with increased stress tolerance of intestinal lactobacilli. Appl. Environ. Microbiol.

[47] Abu Mweis S.S., Jew S., Ames N.P. (2010). β-glucan from barley and its lipid-lowering capacity: A meta-analysis of randomized, controlled trials. Eur. J. Clin. Nutr.

[48] Rieder A., Knutsen S.H., Ballance S., Grimmer S., Airado-Rodríguez D. (2012). Cereal β-glucan quantification with calcofluor-application to cell culture supernatants. Carbohydr. Polym.

[49] Meijerink M., Wells J.M., Taverne N., de Zeeuw Brouwer M.-L., Hilhorst B., Venema K., van Bilsen J. (2012). Immunomodulatory effects of potential probiotics in a mouse peanut sensitization model. FEMS Immunol. Med. Microbiol.

[50] Van Hemert S., Meijerink M., Molenaar D., Bron P.A., de Vos P., Kleerebezem M., Well J.M., Marco M. L. (2010). Identification of *Lactobacillus plantarum* genes modulating the cytokine response of human peripheral blood mononuclear cells. BMC Microbiol.

[51] Sheu B.S., Wu J., Lo C.Y., Wu H.W., Chen J.H., Lin Y.S., Lin M.D. (2002). Impact of supplement with lactobacillus and bifidobacterium-containing yoghurt on triple therapy for *Helicobacter pylori* eradication. Aliment. Pharmacol. Ther.

[52] Macouzet M., Lee B.H., Robert N. (2009). Production of conjugated linoleic acid by probiotic *Lactobacillus acidophilus* La5. J. Appl. Microbiol.

